# Color education: A study on methods of influence on memory

**DOI:** 10.1016/j.heliyon.2022.e11607

**Published:** 2022-11-16

**Authors:** Inna Diachenko, Svitlana Kalishchuk, Mykhailo Zhylin, Andriy Kyyko, Yuliya Volkova

**Affiliations:** a Department of Natural and Social and Humanities Disciplines, “Zhytomyr Medical Institute” of Zhytomyr Regional Council, Zhytomyr, Ukraine; b Psychology of Personality and Social Practices Department, Borys Grinchenko Kyiv University, Kyiv, Ukraine; c Department of Practical Psychology, Odesa National Maritime University, Odesa, Ukraine; d Department of Olympic and Professional Sports, Kharkiv State Academy of Physical Culture, Kharkiv, Ukraine; e Department of Emergency Medicine, Anaesthesiology and Intensive Therapy, Kharkiv National Medical University, Kharkiv, Ukraine

**Keywords:** Color identification, Color memory, Online education, Blended learning, Color-coding

## Abstract

This study examines the mechanisms and expertise of color-based method implementation in a present-day academic process and different forms of learning. This study aimed to identify the effectiveness of color education in the study of the humanities (history of Ukraine) for medical students. The research methodology included structural and logical methods, questionnaire methods, observations, and descriptive and statistical methods. The research results include an identified system of effective parameters, forms, and techniques of color education used in the academic process as well as its impact on the quality of education services provided under blended learning conditions. The color-coding culture parameter color-coding culture of important text segments ranked first among the seven techniques to activate mental activity and memory retention intensification. Color coding has become medical students' most effective method of remembering information. Color-based methods in the teaching of humanities are an effective method for improving the quality of students' learning and allow for better memorization of learning materials, especially in distance learning environments. Prospects for introducing pedagogical innovations in higher education include improving and developing educational materials using color effects to improve student perceptions. The research can be applied to the educational process for students of various specialties and the study of different disciplines.

## 1 Introduction

Identification and inclusion of the psycho-physiological peculiarities of medical students’ perception of learning material through colors remain a live issue today. The paper investigates the impact of the system of color methods, color association methods, and other innovative technologies on the intensification of the cognitive learning process components and responses to the out world (Chaturvedi et al., 2022; Roy et al., 2021).

Cognition implies involvement in several intertwined psychic processes. Memory, attention, mode of thought and perception are closely interrelated, which sets out the success level both of learning processes and innovative advancements in education (Fuchs et al., 2019).

The transition from traditional to distance education forms, including online learning, remains a research issue (Jammula et al., 2021; Arnicane and Souza, 2022). Online learning implies a certain adaptation and transformation process of educational systems, tools, forms, and learning technologies. That is a shift in standards and traditions of the teaching activity, a change of the current thinking paradigm existing at the levels of academic-and-administrative staff, and education seekers. The offered approaches imply various forms of adaptation for education seekers, psychologists, and psychotherapists’ advice, the development of special learning technologies, the development of the system of counseling services, and other measures (Roy et al., 2021).

Seeking to improve distance learning efficiency, the researchers explore the techniques for enhancing various types of memory (Riva et al., 2021; Justus et al., 2021). The levels of attention to specific stimuli increasing the probability of memory retention are being investigated from the viewpoint of the repeated response rate. The information, which is given more attention, increases the probability of memory retention and decreases if the learning material goes beyond a priority area or has been fully ignored (Smilek et al., 2002; Zavaruieva et al., 2022).

Smilek et al. (2002) discussed the potential of vigorous activity to improve short-term and long-term memory, the peculiarities of human memory performance being improved under the conditions of harmonious color against other colors. Zavaruieva et al. (2022) wrote about the influence of colored images and illustrations. Wichmann et al. (2002) stated that the perception of colored pictures and subsequent memory retention was better than with black and white pictures, although the difference was not significant. In addition, attention tasks are better given to students in brighter light, and tasks that need to remember - in less bright light (Castro-Alonso et al., 2018; Mogas-Recalde and Palau, 2021). It is possible to improve the perception of students with lighting (Lekan-Kehinde and Asojo, 2021; Liu et al., 2022), and experts Suh et al. (2020) have researched that color and light have a significant influence on the quality of learning. Considering this, we believe that the color of the materials also has a strong impact on students' productivity.

In a sense, this explains students’ preference for colored images and spending less time recognizing colored objects than colorless images. A colored object against colorless background contributes to better memory retention and ensures a shorter response time as compared to a colored object placed against a colored background (Wichmann et al., 2002; Zavaruieva et al., 2022). The color of educational materials is one of the elements of the learning environment that has an impact on learning outcomes (Luis et al., 2019).

### 1.1 Literature review

Distance learning, its content, essence, and structure have been studied from various perspectives (Riva et al., 2021; Glaser et al., 2022; Falk et al., 2022). Some researchers merge the notion of distance learning with the concept of online education (Fuchs et al., 2019). Several studies also raise concerns about the introduction and implementation of distance education (Riva et al., 2021; Jung et al., 2022; Wagner et al., 2022). The main challenges refer to emotional tension, lack of social interaction, communication problems, low level of digital education, lack of command of time-management basic skills, and weak or wrongful motivation (Saryazdi et al., 2022). This is what makes us undertake a deep analysis of the distance learning system as an indispensable didactic-and-curricular format under pandemic conditions. The studies on modern education problems prioritize the search for ways to improve learning processes and educational innovations which are meant to increase the quality of present-day medical education under pandemic conditions (Dincer and Inangil, 2021). The increase in the education level is also connected to the performance of blended learning and the activation of various types of memory, attention, and emotions (Ghaye, 2010; Riva et al., 2021) and using effective conditions for learning with different devices (Liu and Wang, 2021).

The latest works on psychology, pedagogy, and methods of teaching outline the problems and prospects of the implementation of color education in the academic process. Excitement, in particular, when augmented emotionally, may be instrumental in learning and retaining information within the memory system. The first theories (Farley and Grant, 1976) stated the effect of colors on attention. This conclusion relied on the studies of the processes of cognition and attention and pointed out a better perception of colored presentations. Nolé et al. (2021) explored the positive impact of the color of classrooms (real and virtual) on the quality of learning and the perception of students.

The studies in the field of memory performance (Namaziandost et al., 2018), the ways of its improvement (Fuchs et al., 2019), the perception and fixation of information (Takimoto, 2020), turned out to be valuable for the academic process. It has been considered the role and significance of color, which activate at various levels under various academic conditions, for optimizing the learning process (West and Silberman, 2020). The range of the above-mentioned studies dedicated to the issues of colors and memory explain the empirical results obtained in this field and offer solutions to the difficulties and problems arising in the application of such methods (West and Silberman, 2020).

It has been also established to what degree colors can strengthen the links between memory and emotional excitement (Eysenck and Keane, 2020). The researchers surveyed students and asked them to give their answers about colors they associate with a range of emotions. It found that the majority of participants associated green color with such feelings as calmness, happiness, comfort, hope, and peace. Black was associated with sadness and depression. Therefore, it may be assumed that colors produce the effect of emotional excitement. The pending issues concern the type of emotion and intensity of excitement evoked by a certain color. Llinares et al. (2021) have explored that cold wall colors in training classes have a better effect on concentration and memorization processes than warm colors. Morita & Kambara (2021) investigated that typical colors had better help memorize than blurred. Specialists investigated the presence of relationships between visibility and perceptually homogeneous color space, which can also significantly affect the processes of concentration and memorization (Bruno et al., 2020).

The problems of using color for retaining the learning content and for increasing the level of attention have been studied as well (Pan, 2010). Various factors of positive influence on human memory enhancement and increased in-memory storage and recognition levels were analyzed (Dzulkifli and Mustafar, 2013; Elliot, 2015; Lu et al., 2016).

Attention, from a psycholinguistic perspective, is an important field of research on color association, concentration, and memory performance for the achievement of effective pedagogical results. Numerous studies proved the importance of colors which may bring working memory into operation in the learning process (Cui et al., 2016; Chang et al., 2018). The above-stated studies established that colors play an important role in reducing the cognitive load on education seekers in the academic process. Thus, the recommendations were developed on the use of colorful backgrounds and colored illustrations for the avoidance of cognitive overload and optimization of learning aims. Besides, the methods of color-coding and color signals are also helpful in stressful situations, caused by present-day learning-and-work conditions for medical specialists (Dincer and Inangil, 2021).

### 1.2 Problem statement

Memory improvement depends on the environment predetermined by colors, objects, and shapes. No color would be the most powerful one in terms of its influence on memory because all colors are at work in the perception of distinctive meaningful elements.

The study aims to identify the effectiveness of color education in the study of the humanities (History of Ukraine) for medical students. The hypothesis presupposes that the color methods in the teaching of humanities allow better memorization of educational material, and is an effective method of improving the quality of student learning, especially in the conditions of distance learning.

Given the objective, it is necessary to fulfill the following research tasks:

- to pilot the “History of Ukraine” learning pack (textbook, online courses, materials for self-study, and project work), with the color methods being used;
- to rank the techniques of mental activity intensification due to color coding in the framework of the “History of Ukraine” academic subject;
- to identify the impact of color methods on respondents' qualitative performance among medical students during the application of the color-coded learning pack.

## 2 Materials and methods

### 2.1 Research design

There are three stages to the research's experimentation. The respondents ranked the methods of mental activity intensification in the context of the academic topic "History of Ukraine" in the first and last stages. The first stage clearly defined the criteria to be considered when using color to teach the "History of Ukraine. The criteria were divided into the following sections:

− the reason for using;
− main purposes of color methods;
− principles of digital methods;
− didactic characteristics of visual perception of colors.

In the first stage of the study, the assessment of the increase in mental activity was made using seven methodologies. A color-operated answer, self-instruction, a system of color-coding the text's key passages, self-control of how learning material feed, mind mapping as a tool for optimization, color serving as a point of reference in learning material, and the ability to effectively present learning content were all present.

The respondents gave answers because the sociological survey aimed at obtaining the necessary percentage data (Mallaeva and Turkovskaya, 2017). The respondents' answers were received during 12 h at the instructors’ e-mail addresses.

In the second stage, the assessment of respondents' performance was made (the total average rating of students’ academic results) concerning “History of Ukraine”. The respondents gave their answers voluntarily, and the research group guaranteed the research group would not make the privacy of the survey, or the personal information received public.

The methods of observation and survey were also used in the second stage. The data were collected and analyzed during the research. Later it was used as answers to research questions put forward in the paper. The process of obtaining sociological survey data was similar to the first stage of the study.

Formally, the learning material contained the texts of lectures, instructional and methodological guidelines for self-study activity, illustration material, project proposals, etc. The course was delivered with a focus on the stages in history, highlighted through the lens of prerequisites for the fulfillment of the unique and vital potential of the Ukrainian people. A color palette was used to illustrate the relationship between the historical development of Ukrainian society and the state of medical advancement. The attention was drawn to the value of human life in the hierarchy of social values and the intensity of medical sector development.

The pedagogical guidelines used by the author of the “History of Ukraine” academic subject were as follows:

− the use of the structural and logical methods (focus on the structure as a totality of relations which are invariant under the condition of transformations made to the main body of the course);
− attention to key notional components of the course is concentrated on the “object-property-relation” trichotomy;
− the guiding principles of clustering the material based on the established interrelation, consistency, declaration of continuous development, and rationality.

In the third stage, the questionnaires were distributed, the data were collected, and the respondents’ attitudes to the proposed learning pack, its main components and characteristics and color-coding were measured. The received data were processed and used as a possible framework for establishing the efficiency of the conducted experiment and piloting and as answers to the questions raised in the research.

In the framework of this stage, Cohen's coefficient was also calculated, which enables us to establish the interrelation between values. The calculation was made according to Eq. (1) (McGrath and Meyer, 2006):

(1) d = (*M*_*1*_+*M*_*2*_)/√((*S*_*1*_^2^ + *S*_*2*_^2^)/2),

Where, *M*_*1*_, *M*_*2*_ – means of the first and second parameters; *S*_*1*_, *S*_*2*_ – the mean-square value of the first and second parameters.

When interpreting Cohen's coefficient, it is necessary to consider the strong interrelation between values if they are close to zero; and the absence of the interrelation, when they are close to -1.

To get a whole picture, the research group addressed a range of methodological developments and theoretical studies to resolve the questions raised in the research. The particular interest lies in the use of color methods in education where color is a prime engine for memory improvement and activation of all types of mental activity. The results of the experiment are given as descriptive statistics outputs based on the questionnaire data.

Specific features of the pilot learning pack on History of Ukraine are:

− a well-structured plan based on colored thematic units;
− lecture’s notes styled with diagrams, tables, and cards (in color);
− personalized sections on the establishment and development of the history of Ukraine and the history of Medicine in Ukraine (online presentations, testing, classroom tests in colors);
− the sections of illustration materials (audio-and-video materials, animation).

It enables students to feel the spirit of the age and enhance their knowledge, etc. The options of color-coding are used as learning techniques, particularly, for the notions requiring cognitive efforts, the realization of communicative ideas, and emotional perception.

### 2.2 Sample

Eighty-two first-year bachelor's degree level students specializing in “Medical care” and “Dentistry” (dental hygienist) (Municipal Higher Education Institution “Zhytomyr Medical Institute”, Ukraine) were engaged in the experiment. The main data collection was made from September 2020 to April 2021.

All the subjects gave their voluntary consent to participate in the experiment.

The students were clustered into 4 groups (EG1 – 20; EG2 – 21; CG1 – 20; CG2 – 21). Two groups were selected as experimental ones (EG1, EG2), who were using the author's learning pack “History of Ukraine” in the context of the academic program "History of Ukraine", for its piloting. This subject was chosen to test the effects of color education since it has a humanitarian focus and has no direct bearing on students' future professional abilities. Before the experiment, students engaged in study groups that were organized by the administration for the educational process.

The students specializing in studied in “Medical care” EG 1 – n = 20 (male 11, female 9), and CG1– n = 20, (male 12, female 8), and students specializing in “Dentistry” studied in EG 2 – n = 21 (male 8, female 13), and CG2 – n = 21, (male 10, female 11). Total male n = 41, female n = 41. The age of the students who participated in the study was 17+-1.1 years.

For memory retention and information acquisition during class and self-study, experimental groups employed the color method, interactive learning tools, audio-, and video materials, presentations, and schemes centered upon the color display of materials. The learning process took place under the conditions of blended learning, mainly caused by lockdown restrictions imposed by the pandemic situation in the country.

Other groups were control ones who were studying under the same curriculum but without the author's learning packs on History of Ukraine being used, there was no active color method applied for memory enhancement in the learning process.

The lessons were given to all groups in the first and second semesters in the form of blended learning (partially – classroom activities (37 h), and partially – online activities (44 h)).

### 2.3 Statistical processing

The numeric data obtained from the respondents required additional grouping and processing. Power Microsoft Excel was used for this, which involved the collecting, processing as well as visualization of data in the form of tables and graphics. Excel's functionality was well-suited to our study (Grech, 2018).

### 2.4 Ethical issues

In the framework of the given research, the students specializing in “Medical care” and “Dentistry” took part; therefore, concerning their rights and obligations, the ethical norms in compliance with the rules of the World Medical Association (2020) were observed.

### 2.5 Research limitations

The suggested research has a range of limitations. It is necessary to draw attention to the project's lengthy duration (one academic hour over an academic year), lack of resources, and, in particular, the impossibility of conducting a lengthy and in-depth analysis with focus groups and in-depth interviews. The research limitation is the study of only one subject (History of Ukraine) using color, and experimenting with first-year medical students. To study the effectiveness of the color teaching methods, the subject for experimentation is defined by a humanitarian focus. Color-learning techniques can be used to study many curricular subjects after studying these qualities.

## 3 Results

**Figure 1:**
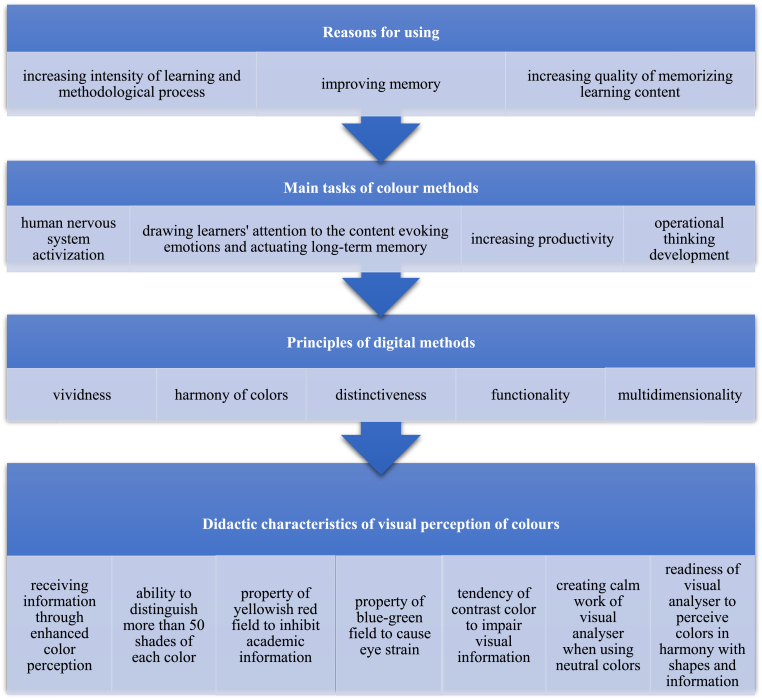
The parameters of using color method fundamentals in teaching the “History of Ukraine”.

“History of Ukraine '' was one of the integrated learning courses implemented under lockdown conditions. In the framework of this course, the textbook based on the color education method was supplemented with online course materials comprising audio content, videos, animation, illustrations, colored and monochromatic schemes and tables, tests, social media facilities, etc. Due to this, it is necessary to determine how important these facilities are to the respondents (see Figure 1).

The main parameters for introducing the color method's foundational concepts in the "History of Ukraine" course were also taken into consideration in the first stage.

In addition, at the first stage, the students evaluated seven techniques of mental activity and memory retention intensification. The researcher offered to rank the parameters set by all the respondents. The results of the ranking were expressed in percentage terms (Table 1).

**Table 1:** Ranking of mental activity-intensification methods within the context of “History of Ukraine” (author's study aid).

Techniques of mental activity intensification	EG1	EG2	CG1	CG2
Color coding system for the important part of the text	24%	39%	32%	25%
The self-control of instructional methods feed	22%	30%	21%	25%
Using mind mapping for optimization	18%	23%	16%	23%
Color serves as a point of reference in educational materials	16%	13%	14%	20%
Self-instruction, a color-operated answer	15%	11%	14%	14%
Skills for effective presentation of educational material	15%	9%	11%	8%

Color coding system for the important part of the text:

● this technique determines what is important and what is secondary in each topic of study;
● there are the key insights when it is important to see and distinguish keynotes;
● the ability to deliver a text in a consistent and structured way results from the ability to establish the level of importance of information with the help of colors, which is an effective method of learning content management.

*The self-control of instructional methods feeds*. It's important to pick the right communication channel and method for distributing learning materials while using blended educational models. That was the goal of the pilot learning course in the experiment's framework. The respondents could watch videos, colored presentations, schemes, and illustrations, and read topic-specific texts.

*Using mind mapping for optimization*: the perception of problems within an academic course is an opportunity to combine multiple perspectives to systematize and present academic material. A person's cognitive abilities are active when they create schemes, presentations, and projects independently (including color schemes).

*Self-instruction, a color-operated answer*: self-instruction aimed at problem-solving within a certain topic, the question based on a large scope of learning material, highlighting important information with color. The algorithm of actions for effective course content mastering is set in a self-instruction manner.

*Color serves as a point of reference in educational materials*. History implies a large amount of information to learn. Assistance in the form of color-coding of various topics, issues, and questions is an effective method of teaching.

*Skills for effective presentation of educational material*. Successful problem-solving techniques for conveying complex information encompassing a large number of facts. The ability to correctly deliver it, activate emotions and attention and make one's work memorable. Based on the obtained results of the survey, it may be stated that the difference in the ranking results for EG and CG at the initial stage is insignificant (Table 1). All the respondents top-ranked the parameter which referred to the culture of color-coding of important segments of a text (30% on average) and the free choice of the learning modes and course content feed (23% on average).

In the second stage, students of all the groups have taught the academic subject “History of Ukraine”. Multidimensionality, structure, and logic in the design of the learning pack based on the color-coding method were the guiding principles for the arrangement of the course content in the experimental group.

The main functions in arrangement and practical use of the learning material within the framework of the course were as follows:

− singling out an element (elements) from the totality of relations;
− seeking particular mechanisms of structural integrity;
− restoring subordinate and coordinate relations between elements, topical units, and clusters;
− consistent analysis of typological properties of structures, and hierarchy;
− revealing fundamental relations within the topic and its immediate scientific value.

The second stage also implied the use of dedicated maps, schemes, and tables. The maps were selected in such a way that the laws of color perception by a visual analyzer were enabled:

− light shades of various colors were used, and each component had its color; the main details, which referred to an object or its parts, were highlighted with color (key parameters) or specific shades of color;
− to assign a separate color to a large scope of a learning object, the active colors of the blue-green field were used; the minor or less important elements were assigned with active colors of the yellowish-red field;
− the profiles were made up of the same color as the surface of a dissected detail.

The tables and schemes are informational media as well, where a certain color-coding regularity is used:

− the contrast between frame line and background; the objects of schemes and tables are placed horizontally; boldface font is used; the correlation between the width of a height letter was 2:3, whereas the correlation of frame to height was 1:6 for creating the effect of direct contrast;
− the most important information was placed in the top left corner and the center of the tables and schemes; the secondary information according to its significance and information was placed in the bottom part and the right bottom corner of the tables and schemes.

The parts of schemes and tables important for memory retention were highlighted with the colors of a yellowish-red field. At the end of the second stage, we made a comprehensive evaluation of the performance indicators of EG and CG, who took part in the experiment. As the results show, the student's performance in EG was higher on average by 11.5% (p < 0.05) (Table 2).

**Table 2:** The qualitative measures of respondents' performance during the implementation of the learning pack with and without color-coding among medical students (author's study aid).

No	Specialty, group	Qualitative indicator of performance in CG	Specialty, group	Qualitative indicator of performance in EG
1	Medical Care CG1	70%	Medical Care EG1	86%
2	Dentistry CG2	67%	Dentistry EG2	74%

At the final stage, all the respondents answered the questionnaire containing the following question: “Which color used in the academic course helps you memorize difficult material better?” The answers required choosing and evaluating colors used in the design of the learning pack materials under the conditions of blended learning. The results are expressed in percentage terms.

**Figure 2:**
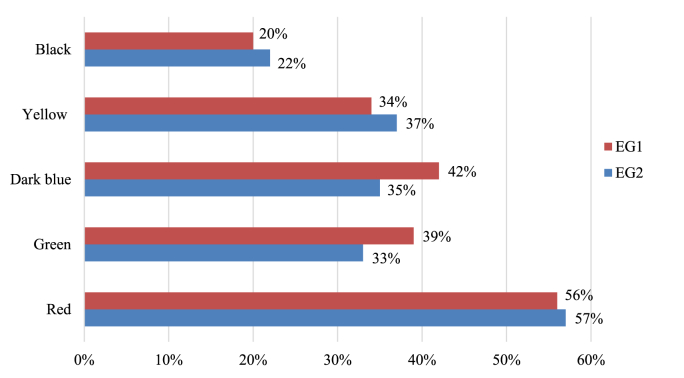
Survey. The color stimulates memory retention (author's study aid).

As follows from the results shown on the bars, the students of the experimental groups noticed the correlation between their perception and a certain color used. The most effective color for memory retention turned out to be red color (EG 1–56%; EG 2–57%), and black color was the least suitable one (EG 1–20%; EG 2–22%) (Figure 2).

In the final stage, a survey on the methods of retaining the content was also conducted. The following task was given to the respondents: “Choose the method you prefer and use for retaining the learning material from the range of offered methods”.

**Figure 3:**
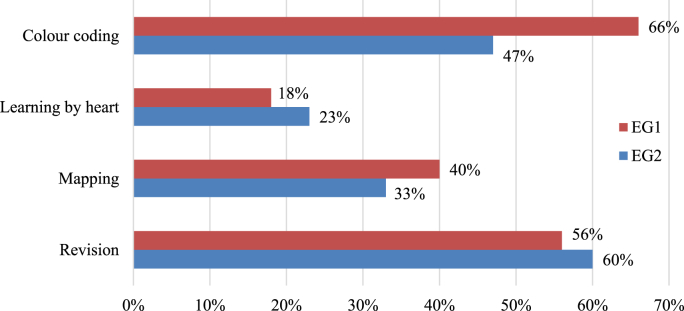
Survey. The methods preferred for retaining the learning material (author's study aid).

As the results of the survey show (see Figure 3), the method of color-coding the information for memory retention is widespread among students (EG1 – 66%; EG2 – 47%). The method of revision also proved to be effective (EG1- 56%; EG2 – 60%), whereas learning by heart was the least effective method for the students who studied History of Ukraine (EG1- 18%; EG2 – 23%).

The research's final stage also included the survey conducted among students concerning the formats for retaining learning material, which was also coded with color in the framework of the pilot academic course. The question was “Which techniques help you retain the learning material?”

**Figure 4:**
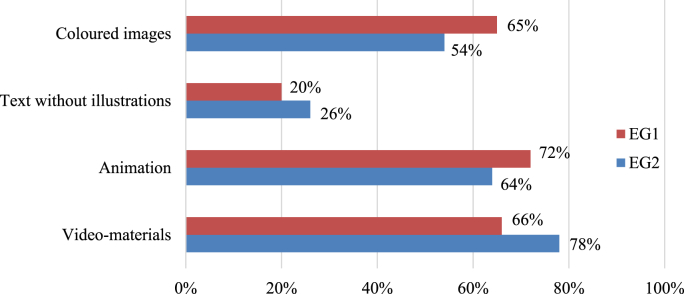
Techniques for better retaining learning material (author's study aid).

As we can see, among the techniques for better retaining learning material, the students prefer video materials (on average 72%), animation (on average 68%), and colored images (on average 55%). These strong indicators demonstrate the value of supplementing academic subjects with the facilities of using color in education, new technologies, and social media (see Figure 4). The opportunities of contemporary education shall not be limited to featureless monochrome texts lacking illustration material, appendices, schemes, and abstractions.

At the final stage, the respondents had to rank the formats for activation of learning activity and memory retention out of seven parameters again. All the respondents ranked the parameters set by the researcher in the survey. The comparison of indicators was made by using Cohen's coefficient according to Eq. (1).

As we can see, the system of color-coding essential information in the text remained to be the main problem. However, in EG, this type of material organization was viewed positively, and its use has increased by an average of 33% (Table 3). The comparison of the data made by using Cohen's coefficient confirms the interrelation between the obtained data within EG1 and EG2 and results within control groups CG1 and CG2, which confirms the effectiveness of this research in the framework of experimental groups.

**Table 3:** The survey on respondents' evaluations of memory activation techniques (author's study aid).

Techniques of mental activity intensification	EG1	EG2	Comparing results of EGs by Cohen's coefficient	CG1	CG2	Comparing results of CGs by Cohen's coefficient
The system of color-coding of the important part of a text.	52%	58%	0.08	33%	28%	0.10
The self-control of the ways of learning material feed.	42%	43%	0.03	20%	25%	0.07
Color is a point of reference in learning material	38%	34%	0.05	18%	20%	0.03
Mind mapping as a means of optimization	23%	25%	0.02	14%	21%	0.23
Self-instruction, a color-operated answer	18%	18%	0.00	15%	17%	0.03
The skills of effective presentation of learning content.	18%	14%	0.02	11%	10%	0.01

## 4 Discussion

The methods of intensification of color-based teaching are being actively developed in contemporary pedagogy (Zavaruieva et al., 2022; Nolé et al., 2021). The classification of emotions based on the criteria of their intensity and corresponding colors was made according to the results of several studies (Jackson et al., 2009). For instance, the researchers revealed that red color is connected to strong emotions, whereas white and gray colors are associated with less intense manifestations and neutral emotions. Such studies prove that color affects emotions and their intensity, and can regulate the degrees of excitement. The degrees of excitement can be modified depending on the emotional component associated with a certain type of color. There are studies on the typology of colors and their influence on attention (Greene et al., 1983). It was established that the colors of the warm scheme (yellow, red, and orange) have a higher impact on attention as compared to cold colors, for instance, brown and gray. In the framework of our research, a range of colors (black, yellow, green, blue, and red) were used in the pilot learning pack for coding and highlighting the content and making illustrations distinct. Respondents noted the existence of a relation between the perception of a certain color (Figure 2): red color was the most helpful for memory retention (EG1 – 56%, EG2 – 57%), whereas black color was the least suitable one (EG1 – 20%, EG2 – 22%). As follows from the results shown on the bars (Figure 2), the students of the experimental groups noticed the correlation between their perception and a certain color used.

The pedagogical studies in the field of development of color methods (Greene et al., 1983) established that warm colors (yellow, orange, red) influence an increase in attentiveness to learning material as compared to cold colors (gray, brown). Instead, studies by Llinares et al. (2021), show that cool colors in classrooms contribute to better concentration and memorization in students. The color of the classrooms and the color of the text are different factors influencing the same qualities (concentration, memorization, reproduction in memory). The colors may also have an emotional effect, which also influences memory retention (Suh et al., 2020). The level and the range of excitement vary according to the emotional component correlating with a certain type of color. It is established that the degree and range of color perception may also depend on the emotional component and the intensity of arousal associated with color perception. Although some studies refute this version (McConnohie, 1999). If anger has an increased capacity for emotional excitement, then the anger-associated color may also have the same influence. For instance, red color refers to strong emotions or other emotions related to other types of colors. As results of the survey show, the method of color-coding the information for memory retention is widespread among medical students (EG1 – 66%; EG2 – 47%), and the method of revision proved to be effective (EG1- 56%; EG2 – 60%), whereas the method of learning by heart was the least effective method (Figure 3).

The exploratory paradigm focuses on the study of the cognitive abilities of students (Dzulkifli and Mustafar, 2013) and the processes of memory retention, the concentration of attention, thinking, etc. Just as Castro-Alonso et al. (2018), Mogas-Recalde and Palau (2021) show the effects of lighting on concentration and memory processes, the use of colors must be justified. However, sometimes important information must be presented in a less bright color in order to activate attention, as this encourages students to look closely at what is written and concentrate. Therefore, the learning process assisting strategies, including color methods, are being actively developed and techniques of using color in teaching students are very wide. They can be used in the academic process in a certain way for enhancing and transforming motivation to study, learn and gain experience. As our study showed, the strategy of the more active use of color methods and making deeper insights into a topic predetermines the prolonged memory capacity. Video materials (on average 72%), animation (on average 68%), and colored images (on average 55%) were named as the most effective techniques of memory retention (Figure 4). This speaks for the importance of color-based methodology in a modern academic process, which shall promote the use of all possible means for easier and more effective learning free from stressors conditions. Methods of activating and improving memory based on the color system, incorporated into the educational process and focused on the development of cognitive and communication skills of the individual, have been proposed for consideration and analyzed in the study of Dzulkifli and Mustafar (2013). Using color for memory stimulation could increase the capacity of coding, storage, and recognition of environmental stimuli, whereas colors and respective manipulations may influence human memory performance (Zavaruieva et al., 2022).

A study of participants' response rates to information presented in color (Hall and Hanna, 2004) concluded that colored objects are perceived, felt, and remembered better compared to the perception of individual shapes of objects of the same color. This result enables us to state that color draws attention during the academic process better than other visual characteristics do. This confirms the research results by Mogas-Recalde and Palau (2021), Liu and Wang (2021) and Bruno et al. (2020). The study of productivity when using the color method in the learning process revealed the results presented in Table 2. In the groups where color was used as a learning method, the performance increased by 11 %, whereas it remained within 2% of probability error in the control groups. Among the memory retention techniques, 55 % of respondents preferred color-coding of important information.

As part of the "History of Ukraine" course, medical students could navigate through the entire course and trace how their priorities in choosing memory techniques changed during the implementation of color-enhancing opportunities (Table 1, Table 3), and evaluate how important such opportunities were to the respondents. The parameter of using color as a reference point in the learning material rose from 5th to 4th place for the experimental groups; scores rose to 36%. The system of color coding of essential information in the text takes first place, in the EG such means of systematizing the material were perceived positively, and the number of those who used such methods increased by an average of 33%.

Based on the results, we can argue that the increase in the volume and methods of respondents' use of color in the learning process indicates a positive assessment of the integrated pilot training package on the history of Ukraine by students. The previously mentioned points out the necessity of using innovative methods and pedagogical technologies in the teaching process. Thus, the hypothesis of the study is confirmed - the use of color in the teaching materials allows for better memorization of the material and contributes to the effectiveness of student learning in general. This is especially important in a distance learning environment given the peculiarities of material perception from digital devices.

## 5 Conclusion

The active use of color in educational activity is a form that enables teachers to encourage their students to express themselves, which, in itself, is a key to their being satisfied with the learning process and its success, as well as with their further career growth. Our study showed that the color-coding system of the important text part proved to be the most significant for students in the experimental groups (24% for EG1 and 39% for EG2). For the students of CG1, the most important is the color-coding system of the important text part, CG2 – color-coding system of the important text part and self-control of the ways of learning material feed, because this contributes to their self-study process.

The student's readiness to use the color method, the availability of specific learning materials and the diversity of communication means in education activate mental activity and improve memory retention. The performance in experimental groups has increased by 11.5% on average, 55% of participants in experimental groups have used the color method in learning, which is 33% higher than it was at the initial stage of the research. This speaks for the appropriateness of using a learning pack supplemented with the methods of color education.

Repeated ranking of the formats of learning activity and memory retention intensification according to seven parameters showed that for the students of experimental and control groups the highest results were obtained for the color-coding system of the important text part: 52% for EG1 and 58% for EG2, 33% for CG1 and 28% for CG2. The comparison of the results by using Cohen's coefficient showed the interrelation of the data between the students of the experimental groups as well as between the students of the control groups.

Color methods applied in the process of teaching the “History of Ukraine” academic subject can be used in pedagogical practice, and are not only implemented in the medical and natural science departments of higher educational institutions but also adapted for humanities and technical specialties.

Further research will include a pedagogical experiment to determine the most effective methods to consolidate and improve students' memory in a distance and blended learning environment. Activating memory based on the emotional perception of colors will unlock the potential to develop independent learning skills and the ability to cope with large amounts of learning material, encouraging students to be purposeful, motivated, and creative in their learning activities.

## Declarations

### Author contribution statement

Inna Diachenko: Conceived and designed the experiments; Performed the experiments; Analyzed and interpreted the data; Wrote the paper.

Svitlana Kalishchuk: Conceived and designed the experiments; Performed the experiments; Wrote the paper.

Mykhailo Zhylin: Contributed reagents, materials, analysis tools or data.

Andriy Kyyko: Performed the experiments; Contributed reagents, materials, analysis tools or data.

Yuliya Volkova: Contributed reagents, materials, analysis tools or data; Wrote the paper.

### Funding statement

This research did not receive any specific grant from funding agencies in the public, commercial, or not-for-profit sectors.

### Data availability statement

Data included in article/supp. material/referenced in article.

### Declaration of interests statement

The authors declare no conflict of interest.

### Additional information

No additional information is available for this paper.
